# Urban gulls adapt foraging schedule to human-activity patterns

**DOI:** 10.1111/ibi.12892

**Published:** 2020-11-02

**Authors:** Anouk Spelt, Oliver Soutar, Cara Williamson, Jane Memmott, Judy Shamoun-Baranes, Peter Rock, Shane Windsor

**Affiliations:** 1 Department of Aerospace Engineering, University of Bristol, Bristol, BS8 1TR, UK; 2 School of Biological Sciences, University of Bristol, Bristol, BS8 1TQ, UK; 3 Institute for Biodiversity and Ecosystem Dynamics, University of Amsterdam, Amsterdam, 1098XH, The Netherlands

**Keywords:** anthropogenic food sources, behaviour, GPS, observations, temporal patterns, urban ecology

## Abstract

Numerous animals are able to adapt to temporal patterns in natural food availability, but whether species living in relatively novel environments such as cities can adapt to anthropogenic activity cycles is less well understood. We aimed to assess the extent to which urban gulls have adapted their foraging schedule to anthropogenic food source fluctuations related to human activity by combining field observations at three distinct urban feeding grounds (park, school and waste centre) with global positioning system (GPS) tracking data of gulls visiting similar types of feeding grounds throughout the same city. We found that the birds’ foraging patterns closely matched the timing of school breaks and the opening and closing times of the waste centre, but gull activity in the park appeared to correspond to the availability of natural food sources. Overall, this suggests that gulls may have the behavioural flexibility to adapt their foraging behaviour to human time schedules when beneficial and that this trait could potentially enable them to thrive in cities.

Optimal foraging theory predicts that animals should adopt a foraging strategy that provides the greatest reward compared with cost, maximizing net energy gain and eventually fitness (Stephens & Krebs 1986). While searching for food, animals have to respond to both spatial and temporal variations in food availability. Some animals are able to adapt to temporal fluctuations in natural resources, many of which vary in predictable ways based on environmental cycles, such as circadian, tidal and seasonal rhythms (Cox *et al*. 2013, Lin *et al*. 2013). In comparison with natural environments, urban environments are novel for animals on an evolutionary time-scale and present a wide array of potential food sources. However, in urban environments, food availability often fluctuates temporally according to anthropogenic activity patterns, such as weekday/weekend cycles. Currently, little is known about how urban animals cope with these fluctuations in anthropogenic food availability.

Readily available food in urban environments is believed to be one of the reasons why numerous animal species are thriving in cities around the world (Shochat 2004). These include insect pollinators (Baldock *et al*. 2015), birds (Marzluff 2001, Chamberlain *et al*. 2009), and carnivorous mammals such as foxes, bears and hyenas (Bateman & Fleming 2012). This increase in urban animals has resulted in complex human–wildlife interactions (Ditchkoff *et al*. 2006), with people either being attractants (a signal that food might be available) or deterrents (causing disturbance). Gulls are an example of species thriving in cities worldwide (Monaghan & Coulson 1977, Balmer *et al*. 2013), but the exact reasons for their success are uncertain and could be a result of several factors such as higher temperatures, fewer predators, ample nesting sites and predictable food conditions (Rock 2005). Gulls exploit numerous anthropogenic food sources, such as food waste and fishery discards (Washburn *et al*. 2013, Tyson *et al*. 2015). They have also been observed to follow fishing vessels during weekdays (Tyson *et al*. 2015) and visit natural and urban feeding grounds at specific times of the day corresponding to their temporal pattern of availability (Sibly & McCleery 1983, Irons 1998, Yoda *et al*. 2012). Using gulls as study species can provide insights into the potential ability of urban animals to adapt their foraging schedules to temporal patterns in food availability.

This study aimed to quantify temporal patterns in gulls’ use of urban feeding grounds and to assess the extent to which gulls have adapted their foraging schedules to human activities. Based on GPS tracking data from a previous study (Spelt *et al*. 2019) we selected three urban feeding grounds frequently visited by the gulls to conduct observations. Given previous observations of the timing of gulls’ use of urban feeding grounds (Yoda *et al*. 2012), we expected the gulls to match their foraging schedule to the times when human activity and/or food availability was highest. Additionally, we predicted that the foraging schedule would vary at each feeding ground, reflecting differences in the temporal characteristics of the food sources.

## Methods

### Study area and species

**Figure 1:**
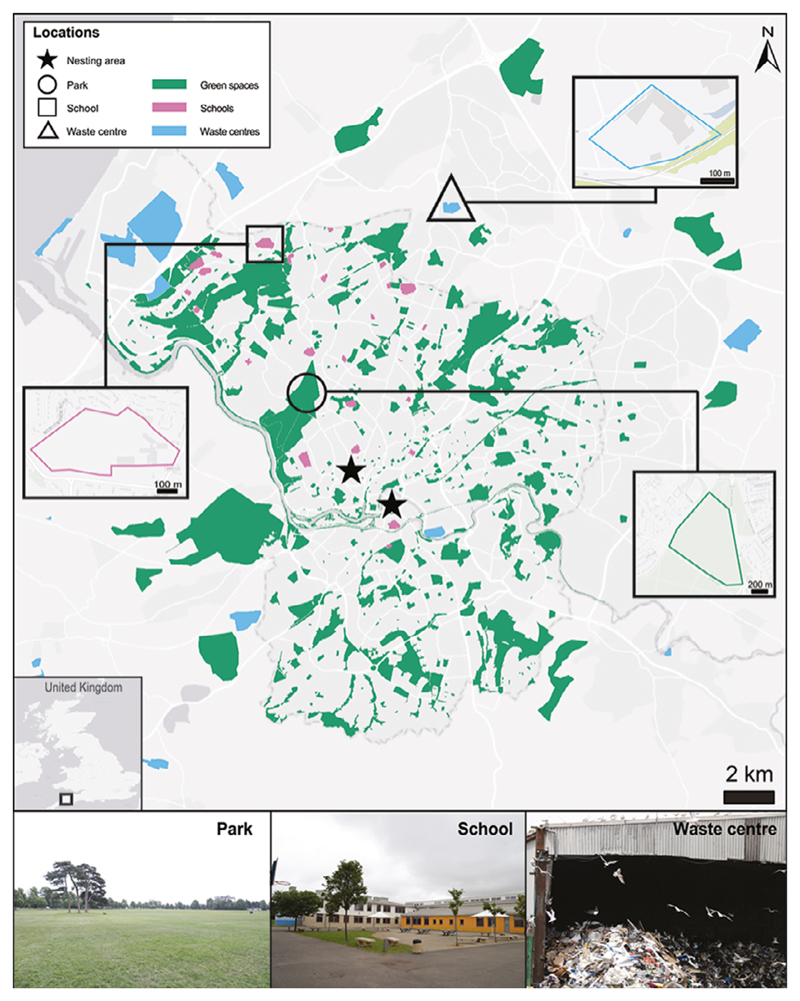
Habitat map of the study area in Bristol, UK, indicating the different habitat types (green spaces, schools and waste centres), the location of the nesting areas (stars) and the specific feeding grounds where ground observations were carried out: the park (circle), the school (square) and the waste centre (triangle). Insets of each specific feeding ground show the area where counts were conducted. Base map sources: ESRI, DeLorme, HERE Technologies, MapmyIndia.

This study was conducted during the gull breeding season between 18 June and 16 July 2018 in Bristol, UK, and was part of a GPS tracking study with Lesser Black-backed Gulls *Larus fuscus* that began in 2016 (Spelt *et al*. 2019). Based on these GPS tracking data, we selected three urban feeding grounds for observations: a park, a school and a waste centre (Fig. 1). These locations were selected because they were frequently used by the GPS-tracked Lesser Black-backed Gulls and were on average 2.9, 6.7 and 7.1 km, respectively, from the two nesting areas (which were ~ 1.5 km apart from each other). Gulls of all species present at these sites were recorded and counted, with no distinction being made between the species. These included Lesser Black-backed Gulls, Herring Gulls *Larus argentatus* and Black-headed Gulls *Chroicocephalus ridibundus*.

### Feeding ground observations

Each of the three feeding grounds was observed for 7 days (chosen at random during the study period). This included two weekend days in order to capture the difference between weekdays and weekends. At each site we conducted counts every 15 min for up to 12 h between 04:00 and 16:00 h (the park), 07:00 and 17:00 h (the school) and 06:00 and 18:00 h (the waste centre). We used the GPS tracking data to identify these locations and the time periods for observation to ensure that the observations included the majority of the time that the gulls were present at these feeding grounds (see Supporting Information Fig. S1). For each count at the park and the school, the following variables were recorded: number of gulls, number of people, anthropogenic food presence and day of the week. Food was considered to be present when people were observed consuming food. For the park, gulls present within the park boundary were included in the counts but gulls flying over the park at high altitudes were excluded. For the school, we counted the gulls present at the school playgrounds, on the surrounding school building and the adjacent sports fields because these areas were all used by people during the day.

The waste centre is a transfer station where 35 000 tonnes of commercial mixed waste, including food waste, is processed annually. At the waste centre, instead of the total number of gulls, we recorded both the number of gulls on the roofs of surrounding buildings as well as gulls on the food waste pile – the distinction being that birds on the food waste pile were actively searching for food, whereas those on the roofs were not. We also recorded the time of any waste-related activity, which was any activity happening on or around the food waste pile, such as unloading food waste. We calculated the time since a waste unload and a waste-related activity level for each count (Supporting Information Table S1). Waste-related activity level measured the level of disturbance and consisted of 0 (nothing happened at the time of the count), 1 (activity off the food waste pile), 2 (single activity on the food waste pile) and 3 (more than one activity on the food waste pile). This resulted in the following variables for the waste centre: total number of gulls, percentage of gulls on the food waste pile, waste-related activity level, time since waste unload and day of the week. For all sites, gull counts were excluded when the gulls were disturbed by birds of prey.

For statistical analysis, we modelled the number of gulls (at the time of each count) in the park and at the school in relation to the following predictors: time of day (continuous – 15 min), number of people (continuous), anthropogenic food presence (categorical – Yes, No) and day of the week (categorical – weekday: Monday–Friday, weekend: Saturday–Sunday). At the waste centre, we modelled the number of gulls (at the time of each count) in relation to the following predictors: time of day, day of the week and waste-related activity level (categorical – four levels: 0, 1, 2, 3). We used generalized additive mixed models (GAMMs) to account for the non-linear relationship between time of day and the number of gulls. Date was included as a random effect to account for multiple observations per day. Lastly, for the waste centre, we also modelled the percentage of gulls on the food waste pile (at the time of each count) in relation to the following predictors: waste-related activity level, day of the week and time since waste unload (categorical – seven levels: 0–15, 15–30, 30–45, 45–60, 60–75, 75–90, > 90 min). We used a generalized linear mixed model (GLMM) because the time of day was not included, as we expected time of day to have no effect on the percentage of gulls on the food waste pile. To this GLMM, we added an offset of the total number of gulls and date was also included as a random effect. Interaction terms of predictor variables were included when this seemed biologically justifiable. Models were created with a negative binomial distribution due to overdispersion. GAMMs were modelled using the mgcv package (Wood 2011) and the GLMM was modelled using the lme4 package (Bates *et al*. 2015) in R version 3.6.0 (R Core Team 2013). For the GAMMs, diagnostics were checked via gam.check and the number of knots was set at the default (*K* = 10). We conducted a forward-step selection procedure to select the ‘best-fit’ model based on chi-square tests (appropriate for negative binomial distributions) following Zuur *et al*. (2009). The fitted models can be found in Supporting Information Table S2. Model residuals were normally distributed and showed homogeneity of variance, and predictor variables did not show collinearity. The significance level was set at α=0.05 and results are reported as the mean and standard deviation.

### GPS tracking data

Global positioning system tracking data collected previously (Spelt *et al*. 2019) were re-examined in this study to compare the observed patterns in foraging schedule based on count data with patterns in site visits based on GPS data. We calculated the percentage of total time spent in the aforementioned types of feeding grounds at specific times of the day, for the same period as the feeding ground observations. For full details of the GPS tracking methods, see Spelt *et al*. (2019). In brief, 12 Lesser Black-backed Gulls in Bristol were tagged with UvA-BiTS GPS devices (Bouten *et al*. 2013) in 2016 and 2017. The weight of both device and harness was 18 g, which was < 3% of the birds’ body mass (mean: 2.4%, range: 2.1–2.7%). These devices recorded location away from the nest at different intervals (from 4 to 300 s), and therefore data were subsampled at a 15-min rate to create equal time resolutions and match the feeding ground observation times. Before the start of this study period, one individual’s GPS device failed, one individual died and one individual did not return to the nesting area. Therefore, data from nine individuals were available for inclusion in this study which had either active or non-active nests. To identify the different feeding grounds in Bristol, a habitat map was created in Arc-GIS Desktop 10.5.1 (Fig. 1). Data were extracted from several datasets: Corine Land Cover European seamless vector database (European-Commission 2016), landfill database (Environment Agency 2019a), allotment database (Environment Agency 2019b) and schools of Bristol dataset (Deepspace Web Services 2019). The final map consisted of three habitat types: green spaces (including parks, allotment sites and sports fields), schools and waste centres. GPS points were selected during 18 June and 16 July 2018 at the same times as the feeding ground observations and resulted in a total of 18 305 GPS points of position inside and outside the nesting area.

First, we identified the percentage of total time spent in the three specific feeding grounds (park, school and waste centre) by dividing the amount of GPS locations in a specific feeding ground by the total number of GPS locations. From the GPS dataset, 18, 44 and 399 GPS points were at the specific park, school and waste centre, respectively, where ground observations were carried out. These specific feeding grounds were visited differently by the nine individuals: four individuals used the park (ID 2: 3x, ID 3: 5x, ID 6: 9x and ID 12: 1x), two individuals used the school (ID 9: 4x and ID 12: 40x) and six individuals used the waste centre (ID 2: 54x, ID 3: 302x, ID 5: 28x, ID 8: 1x, ID 9: 13x and ID 11: 1x).

Secondly, we identified the percentage of total time spent in the three habitat types of interest. From the GPS dataset, 918 GPS points were located in green spaces (~ 150 locations), 185 in schools (25 locations) and 680 in waste centres (49 locations). These habitat types were used by all gulls over the 4-week period. All work was approved by the University of Bristol Animal Welfare and Ethical Review Body (UIN UB/15/069) and access permissions were obtained from all properties visited.

## Results

**Figure 2:**
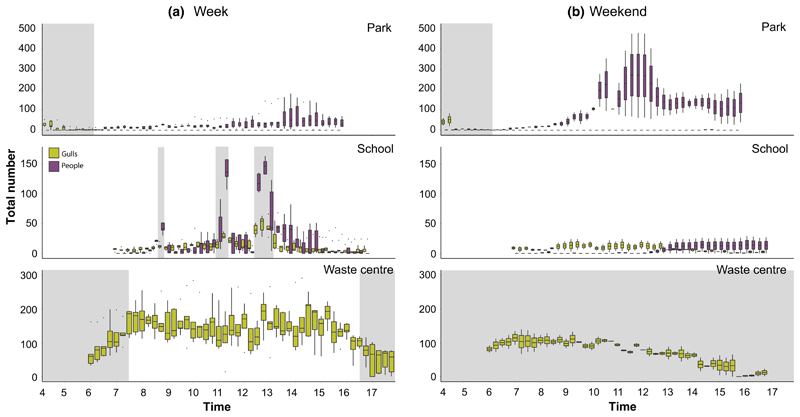
The number of gulls (yellow) and people (purple) based on the feeding ground observations during the week (a) and weekend (b) at the three specific feeding grounds: park, school and waste centre. At the waste centre, the number of people was not counted, but instead waste-related activity level (not shown here, Fig. S4). Grey areas represent the period until sunrise (park), break times (school) and times of closure (waste centre). The boxplots show the 25, 50 and 75% quantiles, the upper and lower whiskers are the largest and lowest value up to 1.5 * inter-quartile range (IQR), and the grey points are data outside 1.5 * IQR.

Based on the feeding ground observations in the park, gulls were mainly present during the early morning when people were not (Fig. 2, Table 1). The number of gulls present was not related to anthropogenic food availability (*χ* ^2^_1_ = 0, *P* = 0.999, see Supporting Information Fig. S2) and there was no difference in the number of gulls present between weekdays and weekends (*χ* ^2^_1_ = 0.444, *P* = 0.657, Fig. 2).

The number of gulls present at the school showed a small peak at 08:45 h (12 ± 5.3) and was highest at 11:15 and 12:45 h local time (25 ± 10.5 and 38 ± 21.5 gulls, respectively), which coincided with an increase in the number of people present (Fig. 2, Table 1) due to the students having breaks at 11:00-11:20 h and 12:20-13:00 h. Additionally, on average significantly more gulls were present when food was present (33 ± 17.4) than when food was not present (9 ± 7.8, Table 1; Supporting Information Fig. S2). Although there were more gulls present during the week (week: 13 ± 10.2 vs. weekend: 8 ± 5.6), this was not statistically significant (*χ* ^2^_1_ = 0.09, *P* = 0.767, Fig. 2), nor was the interaction between time and day of the week (*χ* ^2^_1_ = 0.01, *P* = 0.999). The interaction effect between the day of the week and number of people present was significant, with an increase in the number of people resulting in increased gull numbers during the week but decreased gull numbers during the weekend (Table 1, Fig. 2).

The waste centre was open from 07:30 to 16:30 h on weekdays but was closed over the weekend. During weekdays at the waste centre, the number of gulls was higher (134 ± 59.7) but the percentage of gulls on the food waste pile was lower (32 ± 25%) compared with during the weekend (73 ± 38.31 and 52 ± 26%, respectively; Table 1, Fig. 2; Supporting Information Fig. S3). During the week, the number of gulls increased in the morning and decreased in the afternoon (Table 1, Fig. 2) but during the weekend, fewer gulls were present and the numbers slowly declined (Fig. 2). The waste-related activity level did not affect the number of gulls present (*χ* ^2^_3_ = 2.40, *P* = 0.495, see Supporting Information Fig. S4); however, higher waste-related activity levels resulted in lower percentages of gulls on the food waste pile (Table 1, Supporting Information Fig. S4). Finally, the percentage of gulls on the food waste pile decreased as the time that had elapsed since a waste unload increased (Table 1, see Supporting Information Fig. S5).

**Figure 3:**
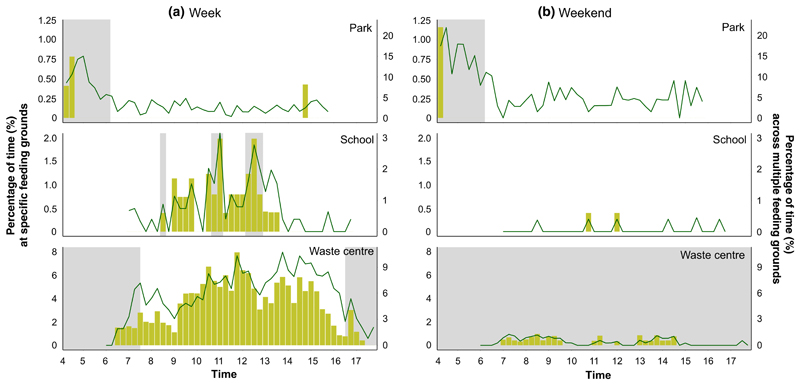
The percentage of total time (%) spent during the week (a) and weekend (b) based on the GPS tracking data. Yellow bars (corresponding to the left *y*-axis) show the percentage of total time at the three specific feeding grounds where ground observations were made: park, school and waste centre. Green lines (corresponding to the right *y*-axis) represent the percentage of time spent across multiple feeding grounds in Bristol, UK: ~ 150 green spaces, 25 schools and 49 waste centres. Grey areas represent the period until sunrise (park), break times (school) and times of closure (waste centre).

The percentage of time the GPS tracked Lesser Black-backed Gulls spent at the three feeding grounds changed over the course of the day (Fig. 3), following similar patterns during the week to those observed in the feeding ground observations (Fig. 2). However, during the weekend, our birds did not visit the three feeding grounds as frequently as during the week, resulting in very low percentages of time in these locations. Additionally, the percentage of time spent at multiple green spaces (including parks), schools and waste centres in Bristol showed that these patterns were not only specific to the three feeding grounds, where field observation were made but were similar for all feeding grounds of these types (Fig. 3 – green line). However, we noted that the temporal pattern at multiple waste centres showed a high peak at the beginning of the day and the temporal pattern at multiple green spaces showed the presence of gulls at later times during the day.

## Discussion

We found that temporal patterns in gulls’ use of urban feeding grounds were specific to each feeding ground, with the park mainly being used in the morning, and the school and waste centre during the day and during weekdays. Our results also show that the temporal patterns in foraging schedule were linked to human activity and food availability. This was mainly evident at the school and the waste centre, where gulls matched their foraging schedule to the times of the school breaks (e.g. a high number of people and presence of food) and times when the waste centre was open (e.g. during the week when waste was unloaded). These results were similar to previous studies where gulls followed temporal patterns of both natural and anthropogenic food sources on a daily scale (Sibly & McCleery 1983, Yoda *et al*. 2012).

The negative relationship between people and gull presence in the park could have been a result of disturbance, as observed in other birds (Fernández-Juricic & Tellería 2000). However, gulls that were present in the morning were predominantly observed walking and pecking for food within the short vegetation (A. Spelt, pers. obs.). Therefore, it seems possible that the presence of earthworms – known to be abundant during early hours of the day (Sibly & McCleery 1983) and to be of great importance for gulls (Coulson & Coulson 2008) – or other arthropods, offers an explanation for the presence of gulls in the morning. This is in agreement with previous studies on foraging behaviour in gulls, where numbers in pasture fields were highest around dawn (Sibly & McCleery 1983) and terrestrial foraging trips were more frequent than marine trips around sunrise (Isaksson *et al*. 2016).

The number of gulls and the number of people at the school were positively related during weekdays, both being more abundant during break times when students were consuming food. However, the relationship was negative during the weekend, indicating that people can act both as attractants (during weekdays) and as deterrents (during the weekend) at the school. However, we must note that these contrasting relationships could be specific to this particular school. Indeed, at the weekend, the sports fields were used by community groups from midday, at which point the gulls, which were present in the morning, were disturbed (A. Spelt, pers. obs.).

At the waste centre, the temporal pattern of the number of gulls present was different during the week and the weekend. During the week, waste was unloaded regularly (up to 15 times a day) during the opening times of the centre. At the weekend, however, no new waste was unloaded due to the centre being closed. This could explain the decrease in the number of gulls with time and the generally lower numbers present at the weekend. These results match those of a study with Herring Gulls where the number of individuals at a refuse tip in Walney, UK, increased when the tip was open and was highest when new waste was unloaded (Sibly & McCleery 1983). The total number of gulls present did not seem to change with activity level, but the percentage of gulls on the food waste pile did decrease. Additionally, the percentage of gulls on the food waste pile was highest just after the waste was unloaded and higher at the weekend when there was no human activity on the food waste pile. These results suggest a possible trade-off between feeding on the food waste pile during an activity, which might be dangerous due to the possibility of injury, and maximizing food intake by foraging when food availability is probably highest.

The percentage of time spent at the three specific feeding grounds, based on GPS tracking data, supported our field observations, showing that the individuals we tracked exhibited similar foraging schedules to the gulls observed during counts following school break times during the week and opening times of the waste centre. Although we only conducted observations at three specific feeding grounds (one site per habitat type), our GPS tracking data demonstrated that the temporal patterns of gull numbers at schools and waste centres are similar across other sites in Bristol of the same habitat type despite the possibility that the exact timing of the gulls’ presence might vary due to different school break times and opening times of the waste centres. Although the GPS tracking data for multiple green spaces showed a similar peak in the morning as found in the observed park, gulls were also visiting green spaces later in the day, possibly attracted by food consumed by people. The observed park is one of the largest parks in Bristol and is used more as a recreational space than as a space to consume food; therefore, this could be a reason that the GPS-tracked gulls did not visit the observed park later in the day.

At both the school and the waste centre, gulls were observed waiting on the surrounding rooftops before school breaks and before waste was unloaded, implying that they were waiting there specifically for food to become available. The temporal predictability of the food sources at these sites appears to have resulted in the birds adopting a sit-and-wait approach instead of actively searching for food (Schoener 1971). This approach may allow them to minimize the time and energy spent searching for food. Similar behaviour has been observed in other bird species. For example, suburban Florida Scrub-Jays *Aphelocoma coerulescens* which had access to predictable human-provided food spent less time foraging and were more efficient foragers than rural Florida Scrub-Jays (Fleischer *et al*. 2003). This suggests that the ability to predict the availability of anthropogenic food sources can maximize net energy gain and fitness, which could eventually be reflected in population growth changes (Stephens & Krebs 1986, Oro *et al*. 2013). It seems that in the present study the availability of food sources is separated in time (park – early morning, school – break times, waste centre – during the day), raising the question of whether the birds are able to optimize their use of resources by tracking their availabilities in a single day. All individuals used all three feeding ground types during the study period, indicating that the individuals do not seem to be specialists and might be able to track resources over a day. More detailed analysis is required to understand this behavioural flexibility and the effects of predicting availability on the birds’ net energy gains.

Numerous animals are able to adapt to natural temporal fluctuations in food availability (Mendes *et al*. 2002, Cox *et al*. 2013, Lin *et al*. 2013) but whether animals are able to cope with anthropogenic temporal rhythms in anthropogenic food availability is relatively unknown. Seabirds have been shown to adjust their foraging strategies to match daily and weekly rhythms in fishery activity (Bartumeus *et al*. 2010, Cama *et al*. 2012, Tyson *et al*. 2015). Although based on a small sample size, we show that gulls in urban environments have the behavioural flexibility to adapt their foraging behaviour to human time schedules by making use of different anthropogenic resources depending on the timings of their availability. These human time schedules differ from natural circadian or seasonal rhythms, as they either happen over shorter time scales (within a day: school break times) or have irregular patterns (weekday vs. weekend: waste centre opening times). This suggests that one of the traits enabling gulls to live so successfully in cities may be their ability to adapt their foraging schedule to human-activity patterns and that this could potentially be a common trait in other successful urban-dwelling species (Bateman & Fleming 2012).

## Supplementary Material

Table S1

Data Set S1-2

Data Set S1-1

Appendix S1

Data Set S1-3

Fig. S1

Data Set S2-1

Table S2

Fig. S2

Data Set S2-2

Fig. S3

Fig. S4

Fig. S5

## Figures and Tables

**Table 1:** Summary of the significance of terms included in statistical models (see Methods) explaining the number of gulls at the different feeding grounds. s(Time), time of day (15 min) as a smooth term; s(Time : week), time of day on weekdays as a smooth term; s(Time : weekend), time of day on weekend days as a smooth term; df, degrees of freedom; edf, estimated degrees of freedom for the smooth term. Estimates (with standard error) are given for the terms in the final best-fit model except for the categorical variables with more than two levels, which can be found in the Supporting Information Appendix S1.

Model	Response	Explanatory	df	*χ* ^2^	*P*	β-coefficients ± se/edf
Park	*Number of gulls (at time of count)*	Number of people (continuous)	1	13.9	< 0.001***	−0.135 ± 0.004
		s(Time)	6.51	46.94	< 0.001***	5.41
School	*Number of gulls (at time of count)*	Number of people (continuous)	1	11.84	< 0.001***	0.0055 ± 0.001
		Food (factor)	1	7.84	0.005**	0.340 ± 0.135
		Number of people: Day of the week	1	4.76	0.029*	−0.057 ± 0.008
		s(Time)	7.08	120	< 0.001***	5.915
Waste centre	*Number of gulls (at time of count)*	Day of the week (factor)	1	241.5	< 0.001***	−0.917 ± 0.189
		s(Time)	5.55	31.768	< 0.001***	4.715
		s(Time : weekday)	4.85	15.695	0.023*	4.086
		s(Time : weekend)	11.92	1.799	0.509	1.468
Waste centre	Percentage on pile *(at time of count)*	Day of the week (factor)	1	14.745	< 0.001***	0.323 ± 0.109
		Waste-related activity level (factor)	3	1601.00	< 0.001***	
		Time since waste unload (factor)	6	241.58	< 0.001***	

*P* < 0.1. ***P < 0.001. **P < 0.01. *P < 0.05.

## Data Availability

The datasets supporting this article have been uploaded as part of the Supporting information Appendix S1.
