# Supplementary material for: Urban gulls adapt foraging schedule to human-activity patterns
Source: Ibis (Lond 1859). Author manuscript; Available in PMC 2021 Jan 1. (PMC7116490; doi:10.1111/ibi.12892)
Supplement: Table S1 [file EMS104399-supplement-Table_S1.doc]

**Supplementary Table 1.** Classification of activity level (AL) for counts at the waste centre.

| **AL** | **Definition** |
| --- | --- |
| 0 | Nothing happened at the time of the count |
| 1 | Activity occurring off the food waste pile at the time of the count, e.g. people walking past |
| 2 | Single activity occurring on the food waste pile at the time of the count, e.g. truck unloading waste |
| 3 | > 1 activity occurring on the food waste pile at the time of the count |
