# Supplementary material for: Urban gulls adapt foraging schedule to human-activity patterns
Source: Ibis (Lond 1859). Author manuscript; Available in PMC 2021 Jan 1. (PMC7116490; doi:10.1111/ibi.12892)
Supplement: Appendix S1 [file EMS104399-supplement-Appendix_S1.docx]

**Supplementary Datasets**

The following datasets were used for analysing the count data at the three specific feeding grounds; park, school, and waste centre. Additionally, we provide the GPS-based dataset used to calculate the percentage of time spent at each of the three specific feeding grounds and at multiple feeding grounds.

**Supplementary Dataset 1-1,**

The raw dataset used for analysing count data at the park. The variables are explained as follows:

• date date when count was made (yyyy/mm/dd)

• time time when count was made (hh:mm:ss)

• gullsTotal number of gulls during count

• peopleTotal number of people during count

• food presence of food during count (yes/no)

• weekday day of the week when count was made (weekday/weekend)

• timeF factor variable of time

• dateF factor variable of date

**Supplementary Dataset 1-2.**

The raw dataset used for analysing count data at the school. The variables are explained as follows:

• date date when count was made (yyyy/mm/dd)

• time time when count was made (hh:mm:ss)

• gullsTotal number of gulls during count

• peopleTotal number of people during count

• food presence of food during count (yes/no)

• weekday day of the week when count was made (weekday/weekend)

• timeF factor variable of time

• dateF factor variable of date

**Supplementary Dataset 1-3.**

The raw dataset used for analysing count data at the waste centre. The variables are explained as follows:

• date date when count was made (yyyy/mm/dd)

• time time when count was made (hh:mm:ss)

• gullsRoof number of gulls on the surrounding roofs during count

• gullsPile number of gulls on the waste pile during count

• gullsTotal um of two previous variables (total number of gulls during count)

• percentagePile number of gulls on the waste pile divided by number of total gulls

• activityLevelF activity level at the moment of the count (factor: 0-1-2-3)

• weekday day of the week when count was made (weekday/weekend)

• timeSinceUnload amount of time since the last waste unload at the count (factor: XS,

S, M, L, XL, XXL, NO)

• timeF factor variable of time

• dateF factor variable of date

**Supplementary Dataset 2-1.**

The dataset used to create the graphical representation of GPS data at the three specific feeding grounds (park, school, waste centre). The variables are explained as follows:

• time time (hh:mm:ss)

• habitat habitat (factor: park, school, waste centre,

other)

• N sample size

• percentage percentage of time spent in habitat during specified time

• sd standard deviation

• se standard error of the mean

• ci confidence interval

**Supplementary Dataset 2-2.**

The dataset used to create the graphical representation of GPS data in the three feeding ground types (parks, schools, waste centres). The variables are explained as follows:

• time time when GPS fix was made (hh:mm:ss)

• habitat habitat where GPS fix was made (factor: green spaces, schools,

waste centres, other)

• N sample size

• percentage percentage of time spent in habitat during specified time

• sd standard deviation

• se standard error of the mean

• ci confidence interval
