# Supplementary material for: Urban gulls adapt foraging schedule to human-activity patterns
Source: Ibis (Lond 1859). Author manuscript; Available in PMC 2021 Jan 1. (PMC7116490; doi:10.1111/ibi.12892)
Supplement: Fig. S1 [file EMS104399-supplement-Fig__S1.docx]

**
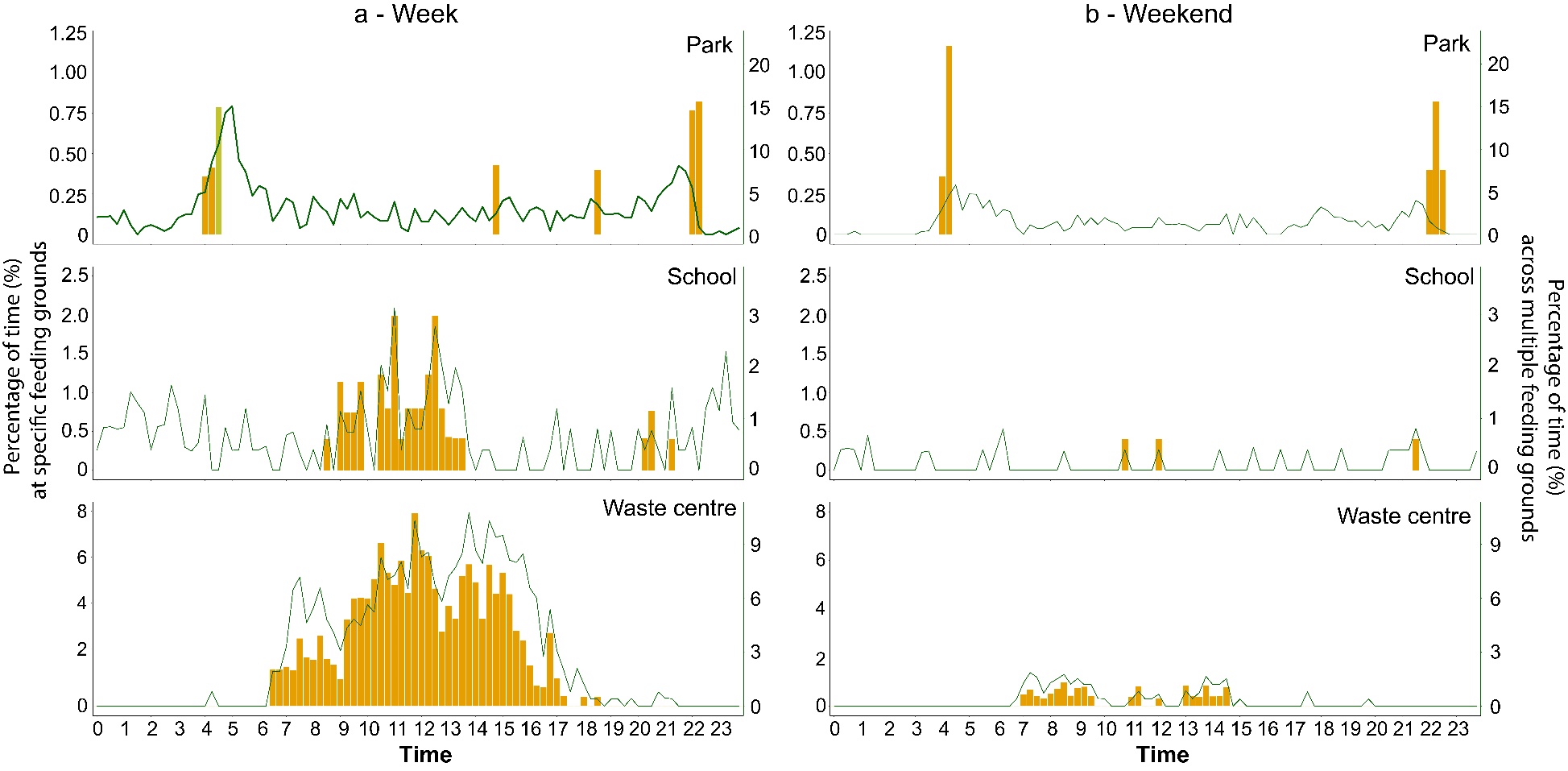
**

**Supplementary Figure 1.** The percentage of total time (%) spent over the 24-hour period during the week (a) and weekend (b) based on the GPS tracking data. Yellow bars (corresponding to the left Y-axis) show the percentage of total time at the three specific feeding grounds where ground observations were made: park, school, and waste centre. Green lines (corresponding to the right Y-axis) represent the percentage of time spent across multiple feeding grounds in Bristol, United Kingdom: ~150 green spaces, 25 schools and 49 waste centres. Grey areas represent the period till sunrise (park), break times (school) and times of closure (waste centre).
