# Supplementary material for: Urban gulls adapt foraging schedule to human-activity patterns
Source: Ibis (Lond 1859). Author manuscript; Available in PMC 2021 Jan 1. (PMC7116490; doi:10.1111/ibi.12892)
Supplement: Table S2 [file EMS104399-supplement-Table_S2.doc]

**Supplementary Table 2.** Overview of the Akaike Information Criterion adjusted for small sample sizes (AICc) used to select the “best-fit” model. The models are 1) the number of gulls in the park, 2) the number of gulls in the school, 3) the number of gulls in the waste centre, and 4) the percentage of gulls on the pile in the waste centre. H = total number of humans, F = presence of food, W = day of the week, AL = activity level, U = time since unloading waste, X*X = interaction between two variables, s(T) = time as a smooth term, df = degrees of freedom, dAICc = difference in AICc between best-fitting model and other models. Grey areas are terms not included in the models.

| **Model** | | **H** | **F** | **W** | **AL** | **U** | **H*F** | **H*W** | **s(T)** | **s(T*W)** | **df** | **AICc** | **dAICc** |
| --- | --- | --- | --- | --- | --- | --- | --- | --- | --- | --- | --- | --- | --- |
| 1 | Park | x |  |  |  |  |  |  | x |  | 15 | 461 | 0 |
|  |  | x | x |  |  |  |  |  | x |  | 15 | 461 | 0 |
|  |  | x |  | x |  |  |  |  | x |  | 15 | 461 | 0 |
|  |  | x |  |  |  |  | x |  | x |  | 15 | 461 | 0 |
|  |  | x |  |  |  |  |  | x | x |  | 15 | 461 | 0 |
|  |  |  |  |  |  |  |  |  | x |  | 12 | 487 | 27 |
|  |  |  |  |  |  |  |  |  |  |  | 2 | 586 | 126 |
| 2 | School | x | x |  |  |  |  | x | x |  | 18 | 1634 | 0 |
|  |  | x | x |  |  |  |  | x | x | x | 19 | 1634 | 0 |
|  |  | x | x |  |  |  | x | x | x |  | 19 | 1636 | 2 |
|  |  | x | x | x |  |  |  |  | x |  | 17 | 1680 | 46 |
|  |  | x | x |  |  |  |  |  | x |  | 16 | 1680 | 46 |
|  |  | x |  |  |  |  |  |  | x |  | 16 | 1685 | 51 |
|  |  |  |  |  |  |  |  |  | x |  | 15 | 1720 | 86 |
|  |  |  |  |  |  |  |  |  |  |  | 8 | 1879 | 246 |
| 3 | Waste centre |  |  | x |  |  |  |  | x | x | 20 | 3296 | 0 |
|  |  |  |  | x | x |  |  |  | x |  | 13 | 3409 | 113 |
|  |  |  |  | x |  |  |  |  | x |  | 13 | 3409 | 113 |
|  |  |  |  |  |  |  |  |  | x |  | 16 | 3414 | 118 |
|  |  |  |  |  |  |  |  |  |  |  | 8 | 3496 | 200 |
| 4 | Waste centre |  |  | x | x | x |  |  |  |  | 13 | 7044 | 0 |
|  |  |  |  | x | x |  |  |  |  |  | 7 | 7272 | 228 |
|  |  |  |  | x |  |  |  |  |  |  | 4 | 8867 | 1823 |
|  |  |  |  |  |  |  |  |  |  |  | 3 | 8879 | 1836 |
