# Supplementary material for: Urban gulls adapt foraging schedule to human-activity patterns
Source: Ibis (Lond 1859). Author manuscript; Available in PMC 2021 Jan 1. (PMC7116490; doi:10.1111/ibi.12892)
Supplement: Fig. S2 [file EMS104399-supplement-Fig__S2.docx]

**

**

**Supplementary Figure 2.** The total number of gulls when food is not present (purple) and when food is present (blue) at the park and the school. Significantly different groups are represented by stars. The boxplots show the 25%, 50% and 75% quantiles, the upper and lower whiskers are the largest and lowest value up to 1.5 * inter-quartile range (IQR), and the grey points are data outside 1.5 * IQR.
