# Supplementary material for: Urban gulls adapt foraging schedule to human-activity patterns
Source: Ibis (Lond 1859). Author manuscript; Available in PMC 2021 Jan 1. (PMC7116490; doi:10.1111/ibi.12892)
Supplement: Fig. S3 [file EMS104399-supplement-Fig__S3.docx]

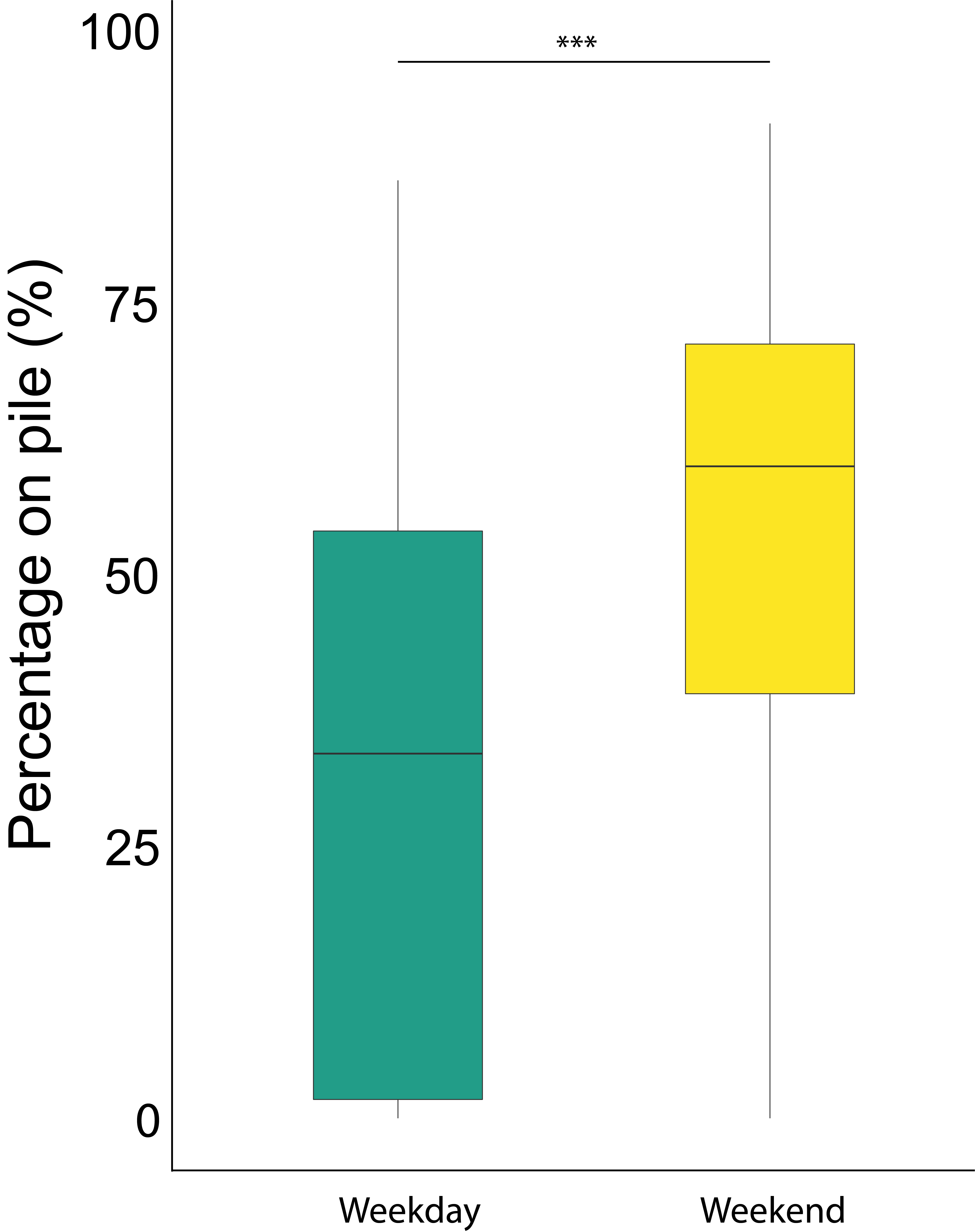


**Supplementary Figure 3.** The difference in percentage of gulls on the waste pile between week and weekend. Stars represent significant differences between groups. The boxplots show the 25%, 50% and 75% quantiles, the upper and lower whiskers are the largest and lowest value up to 1.5 * inter-quartile range (IQR), and the grey points are data outside 1.5 * IQR.
